# Swift microbiome‐mediated phenotype transfer from transgenic plants

**DOI:** 10.1002/jeq2.70070

**Published:** 2025-08-15

**Authors:** Ferran Garcia‐Pichel, Júlia Farias, Vanessa Fernandes, Daniel Roush, Tami L. Swenson, Suzanne M. Kosina, Trent R. Northen, Huansheng Cao, Samual Jaunin, Raju Kandel, Roberto Gaxiola

**Affiliations:** ^1^ School of Life Sciences Arizona State University Tempe Arizona USA; ^2^ Center for Fundamental and Applied Microbiomics, Biodesign Institute Arizona State University Tempe Arizona USA; ^3^ Environmental Genomics and Systems Biology Division Lawrence Berkeley National Laboratory Berkeley California USA; ^4^ The DOE Joint Genome Institute Lawrence Berkeley National Laboratory Berkeley California USA

## Abstract

The expression of an organism's genes determines its own characteristics in any given environment. In this study, we demonstrate that the phenotypic traits of genetically modified transgenic *Arabidopsis thaliana* plants, designed for nutrient efficiency and enhanced yield, can be naturally and readily transferred to neighboring wild‐type plants. Our findings reveal that the transgenic plants significantly influence the populational, compositional, and functional traits of their root‐associated microbiome (RAM), resulting in a larger population, with distinct composition and high functional potential compared to wild‐type plants, regardless of soil type. This phenomenon appears to stem from altered metabolite exudation patterns, which enhance root recruitment. Notably, the RAM plays a dual role: it not only contributes to the robust phenotype of the transgenic plants but also facilitates the transfer of these traits to adjacent wild‐type plants. Upon transplanting wild‐type plants into the presence of transgenics, we observed the induction of transgenic‐like phenotypes. Metagenomic and compositional analyses indicate that this transfer is linked to an increase in 2,3‐butanediol (2,3‐BD) fermenting bacteria. Furthermore, exposure to 2,3‐BD alone was sufficient to elicit transgenic phenotypes in wild‐type plants. These results suggest that factors external to plant tissues, such as root‐associated bacteria and their volatile metabolic products, play a crucial role in the transferability of plant phenotypes to neighboring plants. Our findings underscore the importance of evaluating microbiome interactions in the context of transgenic organisms and open new avenues for alternative agricultural practices that may reduce reliance on genetic modification.

## Core Ideas

- Transgenic plants can enhance nutrient efficiency and yield while transferring traits to neighboring wild type.
- Root metabolite exudation boost microbial abundance and diversity, reshaping the root‐associated microbiome.
- Root‐associated microbiome mediates phenotype transfer between transgenic and wild‐type plants.
- Increased 2,3‐butanediol links transgenic‐induced phenotypic changes to microbial interactions.
- There is potential for microbiome‐based strategies to improve agricultural resilience.

## Plain Language Summary

This study shows that the traits of genetically modified *Arabidopsis thaliana* plants, which are designed to be more efficient in using nutrients and produce higher yields, can be passed on to nearby wild plants. The modified plants change the makeup and function of the bacteria in their root environment, leading to a larger and more diverse microbiome compared to the wild plants. This change is linked to different substances released by the roots, which attract more beneficial bacteria. Interestingly, when wild plants are grown near the modified ones, they start to exhibit similar traits. The research found that this trait transfer is associated with an increase in certain bacteria that produce a compound called 2,3‐butanediol (2,3‐BD). Just exposing wild plants to 2,3‐BD can also make them take on traits of the modified plants. The findings suggest that understanding these interactions could lead to new agricultural methods that reduce the need for genetic modifications.

**Abbreviations**
2,3‐BD2,3‐butanediolASV: 2,3‐butanediolASVamplicon sequence variant
BRAM: bulk root‐associated microbiome
Col‐0: Columbia‐0 (wild‐type Arabidopsis thaliana ecotype)
H^+^‐PPase: proton‐pumping pyrophosphatase
MS: Murashige and Skoog
qPCR: quantitative real‐time PCR
RAM: root‐associated microbiome

## 1 INTRODUCTION

Genetic modification of crop genotypes for desired phenotypes is standard in agriculture (Klümper & Qaim, 2014). The correspondence between genotype and observed phenotype (Johannsen, 1911) is implicitly assumed to be universal, after accounting for environmental effects. One such type of genetic modification involves the plant gene for type I H^+^‐pyrophosphatases, a yield enhancement determinant whose overexpression triggers production of more, larger leaves and root biomass over the wild type (Gonzalez et al., 2010; J. Li et al., 2005), likely due to enhanced photosynthate transport from source to sink tissues such as roots, which in turn promotes nutrient and water use efficiency (Bao et al., 2008; Schilling et al., 2014) in *Arabidopsis thaliana*.

Similar differential phenotypes of proton‐pumping pyrophosphatase (H^+^‐PPase) overexpressing transgenics are seen in crop plants including rice, barley, tobacco, cotton, alfalfa, maize, and wheat (Gaxiola et al., 2016). Another very conserved phenotypic trait associated with these genetically modified plants is an increased acidification of the soil surrounding roots (Bao et al., 2008; Pizzio, Paez‐Valencia, et al., 2015; Yang et al., 2007).

Because all the differential phenotypic traits of these transgenics are principally governed by plant physiological mechanisms, one should expect that their expression is constrained to the plants bearing the specific genetic modifications that elicit them. We were surprised to observe that wild‐type *A. thaliana* plants showed phenotypes resembling those of high‐yield transgenic mutants when they were grown as neighbors of the transgenics and initiated an investigation of this phenomenon, the results of which we present here.

This observation implicates factors external to the plant as determinant of phenotype. Several indirect lines of evidence point to the root‐associated microbiome (RAM) as a suspect. First, the potential for plant‐root microbiome interactions in determining outcomes is becoming apparent (Berg & Smalla, 2009; Schlaeppi & Bulgarelli, 2014), and evidence exists for the potential of microbiome compositional blending in mixed cropping of plant varieties (Horner et al., 2019). Second, the enhanced photosynthate transport to roots, and the acidification of the soil surrounding the root system, typical of the mutant plants, are factors known to affect soil microbiomes: pH is the most important environmental factor driving soil microbial diversity (Fierer & Jackson, 2006; Song et al., 2022), and long‐standing principles (Hiltner, 1904) predict that increased root exudation will result in higher microbial biomass.

Modifications in exudate composition in fact are known to shift RAM composition (Bais et al., 2006; Lebeis et al., 2015). Even genetically close accessions of *A. thaliana* (Monchgesang et al., 2016) and crop plants (Horner et al., 2019) sustain distinct RAMs. As the global population continues to grow, the impending constraints of water and nutrient availability pose significant challenges for food production. In this context, microbiome‐based approaches are being explored as a promising strategy for sustainable agriculture. The concept of plant‐mediated indirect selection of rhizospheric microbiomes offers the potential to cultivate robust and efficient microbial communities that can enhance desired plant phenotypes and overall fitness. This “next‐generation biological” strategy aims to optimize plant‐microbe interactions, ultimately contributing to improved agricultural resilience and productivity (Dubey & Sharma, 2021; Kumar et al., 2024).

A study revealed that overexpressing SWEET transporters in the root epidermis can significantly boost sugar secretion, with SWEET1 enhancing glucose release and SWEET11 promoting sucrose secretion. This increase in sugar not only elevates microbial abundance relative to root weight but also alters the RAM composition (Song et al., 2022). Notably, SWEET1‐overexpressors attracted beneficial bacteria like Rhodanobacter, known for enhancing stress tolerance and disease resistance, while SWEET11‐overexpressors saw an increase in Chitinophagaceae, which may possess pesticide properties. Interestingly, applying sucrose directly to the soil did not replicate these microbiome changes, highlighting the importance of root‐secreted sugars in shaping microbial interactions (Song et al., 2022).

Moreover, in some cases (Chen et al., 2020; O'Brien et al., 2021; Ritpitakphong et al., 2016), traits that are conferred to plants by microbial partners can be artificially transferred by microbiota transplantation. On this basis, we hypothesized that part of the phenotypic robustness shown by our transgenics could be traced back to a more densely populated or functionally modified RAM.

## 2 MATERIALS AND METHODS

### 2.1 Experimental design

The general experimental design included a quantitative demonstration of the initial qualitative observations of plant phenotype transfer. This was carried out in controlled growth experiments using variations of the genotype of neighboring plants. The hypothesized impact of the microbiome was addressed using populational and compositional assessments of the RAMs, and the monitoring of plant phenotypes in the presence or absence of root microbiomes with imposed root microbiome transplants. Probing of the mechanisms of interaction leading to this phenomenon used characterization of plant root exometabolomes and root microbiome metagenomes. Because of variability, biological replication was high (i.e., *n* > 8 individual plants), and the exact level of biological replication given was appropriate for each experiment. These approaches are detailed below.

### 2.2 Plant lines

Genetically modified plant lines were available from prior research. Briefly, “Col‐0” (Columbia‐0 [wild‐type Arabidopsis thaliana ecotype]) was the wild type, “*AVP1*” presents ubiquitous overexpression of the *AVP1* proton pyrophosphatase through insertion of a *35S::AVP1* cassette (Gaxiola et al., 2001), and *pCoY* presents phloem‐specific expression of the proton pyrophosphatase through insertion of a [*pCoYMV]::AVP1* cassette endowed with the Commelina Yellow Mottle virus promoter (Pizzio, Paez‐Valencia, et al., 2015).

**Core Ideas**

- Transgenic plants can enhance nutrient efficiency and yield while transferring traits to neighboring wild type.
- Root metabolite exudation boost microbial abundance and diversity, reshaping the root‐associated microbiome.
- Root‐associated microbiome mediates phenotype transfer between transgenic and wild‐type plants.
- Increased 2,3‐butanediol links transgenic‐induced phenotypic changes to microbial interactions.
- There is potential for microbiome‐based strategies to improve agricultural resilience.

### 2.3 Plant growth

All seeds were surface sterilized by immersion in a solution of 2% sodium hypochlorite and 0.01% Tween 20 for 10 min, followed by three rinses in sterile deionized water (2 min each). Prior to sowing in natural soil, seeds were stratified in the dark at 4°C for 24 h. Sowing was performed in round plastic pots (6 cm diameter × 5 cm height), which were spatially randomized and placed in growth chambers set to short‐day conditions: 8 h of light (300–400 lx) at 21°C, followed by 16 h of darkness at 18°C. Soil moisture was maintained by biweekly irrigation with deionized water, using a scale to restore pot weight to approximately 80% of field capacity.

During the early seedling stage, plants received a one‐time application of half‐strength Murashige and Skoog (MS) medium (GIFCO BRL), pH 5.8, without vitamins. For plate‐based growth, seeds were sown on agar or agarose (0.9% w/v) containing half‐strength MS medium supplemented with 1% (w/v) sucrose. Plates were stratified as described above, then incubated at a 45° angle for 1 week. Seedlings were subsequently transferred to sucrose‐free plates and grown for an additional 2 weeks.

For artificial soil experiments, seedlings from agar plates were transplanted into either top‐open square plates (100 × 100 × 15 mm, crystal‐clear polystyrene) or sealed plastic boxes (under non‐sterile or sterile conditions, respectively), each filled with a compacted substrate and incubated under the same growth chamber conditions described above.

The soils used in this study included

1. Arizona soil – a locally sourced aridisol‐derived garden soil (170 g per plate, 90 g per pot);
2. Iowa soil – a dark organic agricultural soil (170 g per plate, 90 g per pot); and
3. Artificial soil – a porous, inorganic ceramic substrate (Turface) with no detectable native soil microbiome (80 g per plate, 45 g per pot).

### 2.4 Acidification assay

The acidification assay was performed according to previous studies (Pizzio, Regmi, et al., 2015). Seeds were germinated and grown aseptically on vertical agar plates for 7 days. Then, seedlings (10 per point) were transplanted to flasks with 4 mL of liquid media (half strength MS, 1% (w/v) sucrose) and were grown for 2 weeks. Roots were washed with the assay solution (quarter strength MS and 2 mM MES [2‐(N‐morpholino) ethanesulfonic acid] buffer pH 6.8) for 5 min. An acidification assay was performed in fresh assay solution through a 12‐h dark/12‐h light cycle. Protons released relative to fresh root biomass were derived from pH measurements so that the assessment is inclusive of all exudate contributions to acidity, although they are expressed as proton equivalents.

### 2.5 Plant harvesting, RAM fractionation, and RAM transplants

For plant biomass determinations, shoots and roots from 5‐week‐old plants were harvested, separated, cleaned, and dried at 60°C for gravimetric determination of dry biomass data. For RAM fractionation and isolation, roots were harvested, and the soil was divided into three distinct fractions as previously described (Lundberg et al., 2012): loose soil, bulk soil, and rhizosphere soil. Loose soil was left behind on the pots on harvesting and discarded.

Bulk, root‐associated soil was manually removed from the roots by kneading and shaking with sterile gloves (sprayed with 70% ethanol) and by patting roots with a sterile (flamed) metal spatula. The rhizosphere fraction was obtained by placing roots freed of bulk soil in a sterile 50‐mL tube containing 25 mL phosphate buffer (per liter: 6.33 g of NaH_2_PO_4_.H_2_O, 16.5 g of Na_2_HPO_4_ 7H_2_O, and Tween 20 [0.01% v/v]), and vortexing at maximum speed for 15 s. This process was repeated until the roots were completely clear. The turbid solution was gravity filtered through a glass funnel with a filter paper into a new 50‐mL tube to remove broken plant parts and large sediments. The turbid solution was then centrifuged for 15 min at 5000. After discarding the supernatant, the rhizosphere pellet was stored at −20°C until further processing. For microbiome transplant experiments, rhizosphere microbiomes from the appropriate plants (either *AVP1* or Col‐0) were fractionated and collected exactly as described above. The microbiome collections were resuspended in 10 mL of saline buffer; immediately thereafter, the optical density of the suspensions was determined at 600 nm in a spectrophotometer and diluted appropriately to a common value of 0.6. Note that 1‐mL aliquots of these suspensions were dispensed onto a single spot of artificial soil pots, and the spot was marked by inserting a sterile toothpick. Wild‐type seeds were then planted on those spots.

### 2.6 Root exudate collection and metabolomics

After 4 weeks growing in agarose MS nutrient media, collected root exudates were filtered using nylon filters of pore size 0.45 µm (Millipore) to remove root sheathing and cells and stored at −80°C until further analyses. Sterile techniques were used throughout, and there was no evidence of contamination in the media. Exudates (3 mL) were placed in 50‐mL tubes, frozen, and then lyophilized. Dried exudates were resuspended in 1 mL mass spectroscopy grade methanol (Honeywell Burdick & Jackson), vortexed for 10 s, sonicated for 10 min, and then centrifuged for 5 min at 3200 × *g*.

The supernatant was collected in 1.5 mL microcentrifuge tubes and then dried down with a Savant SpeedVac SPD111V (Thermo Scientific) for 1 h with a final resuspension in ice‐cold methanol containing 13C–15N amino acid internal standards (200 µL) and filtration through 0.22‐µm centrifugal membranes (Nanosep MF, Pall Corporation) by centrifuging at 10,000 × *g* for 5 min. Samples (50 µL) were transferred to liquid chromatography‐mass spectrometry vials for metabolomics analysis, using hydrophilic interaction liquid chromatography for metabolite separations and electrospray ionization tandem mass spectrometry for metabolite detection. Chromatographic separations were performed with a SeQuant ZIC‐HILIC 200Å, 5 µm, 150 × 2.1 mm column (1.50454.0001, MilliporeSigma) on an Agilent 1290 series ultra‐high performance liquid chromatography as previously described (Porcar et al., 2018). MS1 and MS2 data were collected as previously described (Porcar et al., 2018). Briefly, metabolites were eluted using a gradient of 5 mM ammonium acetate in water (A) and 5 mM ammonium acetate in 95% v/v acetonitrile in water (B) as follows with a 0.45 mL/min flow rate: 1.5 min at 100% B, 13.5 min linear gradient to 65% B, 3 min linear gradient to 0% B, 5 min at 0% B, 2 min linear gradient to 100% B, and 5 min at 100% B; eluted metabolites were detected using a Thermo Q Exactive hybrid Quadrupole‐Orbitrap Mass Spectrometer equipped with a heated electrospray ionization source probe (Thermo Scientific) using data‐dependent MS2 Top2 function with an *m*/*z* range of 70–1050 and stepped collision energies of 10, 20, and 30.

Metabolomics data were analyzed using Metabolite Atlas with in‐house Python scripts to obtain extracted ion chromatograms and peak heights for each metabolite (Bowen & Northen, 2010); python code is available at https://github.com/biorack/metatlas. Metabolite identifications were verified by comparison with authentic chemical standards and validated based on three metrics: accurate mass (<5 ppm for positive ion mode, <20 ppm for negative ion mode), retention time within 0.5 min of prediction, and MS/MS fragmentation spectra similarity. Data from internal standards and quality control samples (included throughout the run) were analyzed to ensure consistent peak heights and retention times. Key for accessing metabolomics LCMS raw and processed data are shown in Figure S5.

### 2.7 DNA extraction, 16S rRNA copy determination, library preparation, and sequencing

The DNA from 0.25 g of soil from each fraction was extracted using the Qiagen Power Soil DNA extraction kit. After fluorometric determination of DNA concentration in the extract (Qubit, Life Technologies), we used qPCR (quantitative real‐time PCR) with a universal 16S rRNA gene primer set to determine 16S rRNA gene number as previously described (Couradeau et al., 2019). The V3–V4 variable region of the 16S rRNA gene was targeted for amplification and Illumina sequencing as previously described (Roush et al., 2018) and run commercially. Raw sequence data were submitted to NCBI and are publicly available under BioProject ID PRJNA594377.

### 2.8 Bioinformatic analyses and phylogenetic assignments

The raw FASTQ file was demultiplexed within the MiSeq Illumina workflow under default parameters. Paired sequences were demultiplexed and analyzed via Qiime 2.10 (Bolyen et al., 2019), using the DADA2 plugin (Callahan et al., 2016) with the following parameters: trunc_len_f:265, trunc_len_r:245, trim_left_f:17, and trim_left_r:20, to resolve amplicon sequence variants (ASVs). For diversity analysis, we followed (40). Briefly, ASVs were aligned with MAFFT7, and a phylogenetic tree was constructed using FastTree Diversity metrics were calculated using the core‐metrics‐phylogenetic plugin. ASVs were classified using a naïve‐bayes classifier trained on the Green Genes 13_8 reference database and using the classify‐sklearn plugin (https://github.com/qiime2/q2‐feature‐classifier).

Feature tables and diversity metrics were then exported, and differential abundance analysis was conducted within QIIME1 using the plugin differential_abundance.py and the DESeq2 algorithm. Principal coordinate analyses were created using the vegan package and graphics were made using R and the ggplot2 package.

### 2.9 Metagenomics

DNA extracts from rhizosphere RAMS of AVPI and Col‐0 plants (three plants each) grown in artificial soil were obtained as above and sequenced independently. Paired‐end raw reads were filtered with a quality score of 25 and assembled using MEGAHIT 1.2.9 (D. Li et al., 2015). Scaffolds shorter 500 bp were discarded. Genes were predicted using Prodigal 2.6.3 (Hyatt et al., 2010). Unique genes were identified with CD‐HIT 4.6.8 with an identity of 95% (Li & Godzik, 2006).

The abundance of individual genes was estimated by re‐mapping the clean reads to the unique genes with RSEM using Bowtie2 as an aligner (Li & Dewey, 2011). Gene functions were annotated with Megan Community version 6.0, with eggNOG, SEED, and Gene Ontology references (Huson et al., 2011). The abundance of particular genes was standardized against the total number of reads in each sample. Ra Raw sequence data were submitted to NCBI and are publicly available under BioProject ID PRJNA646933.

### 2.10 Statistical analyses

All statistics were performed within the R statistical environment, version 4.0.0. To test hypotheses in plant phenotype comparisons, we used single‐factor ANOVA, followed by post hoc *T*‐test corrected for multiple comparisons, as supported by Excel, whenever more than two treatments were being compared. When using single factor ANOVA, we followed by post hoc *T*‐test corrected for multiple comparisons with Tukey's Honestly Significant Difference (HSD). When only two treatments were compared, we used simple two‐tailed *T*‐tests. The levels of true replication (*N*) and the levels of significance (*p*) are given explicitly for each experiment where applicable, either in the text or figure legend. For microbiome community composition, community differences were assessed via permutational multivariate analysis of variance, performed on Bray–Curtis distance matrices of relative abundances derived from sequencing and using 9999 permutations.

Microbial community differences were visualized using principal component analysis. The relative abundance of genes or groups of genes of interest was compared between the wild type and mutant RAMS using *t* test in R(34), with data logit transformed as typical for proportion data. For metabolomics, statistical comparisons (ANOVA and Tukey HSD, *α* = 0.05) and heatmaps (peak heights scaled across samples relative to max for each compound; rows and columns clustered using complete clustering method with Euclidean distance measure) were generated in R using multcomp (Hothorn et al., 2008) and pheatmap (Kolde, 2012) packages, respectively.

## 3 RESULTS AND DISCUSSION

### 3.1 Phenotype transfer to neighboring plants

**FIGURE 1:**
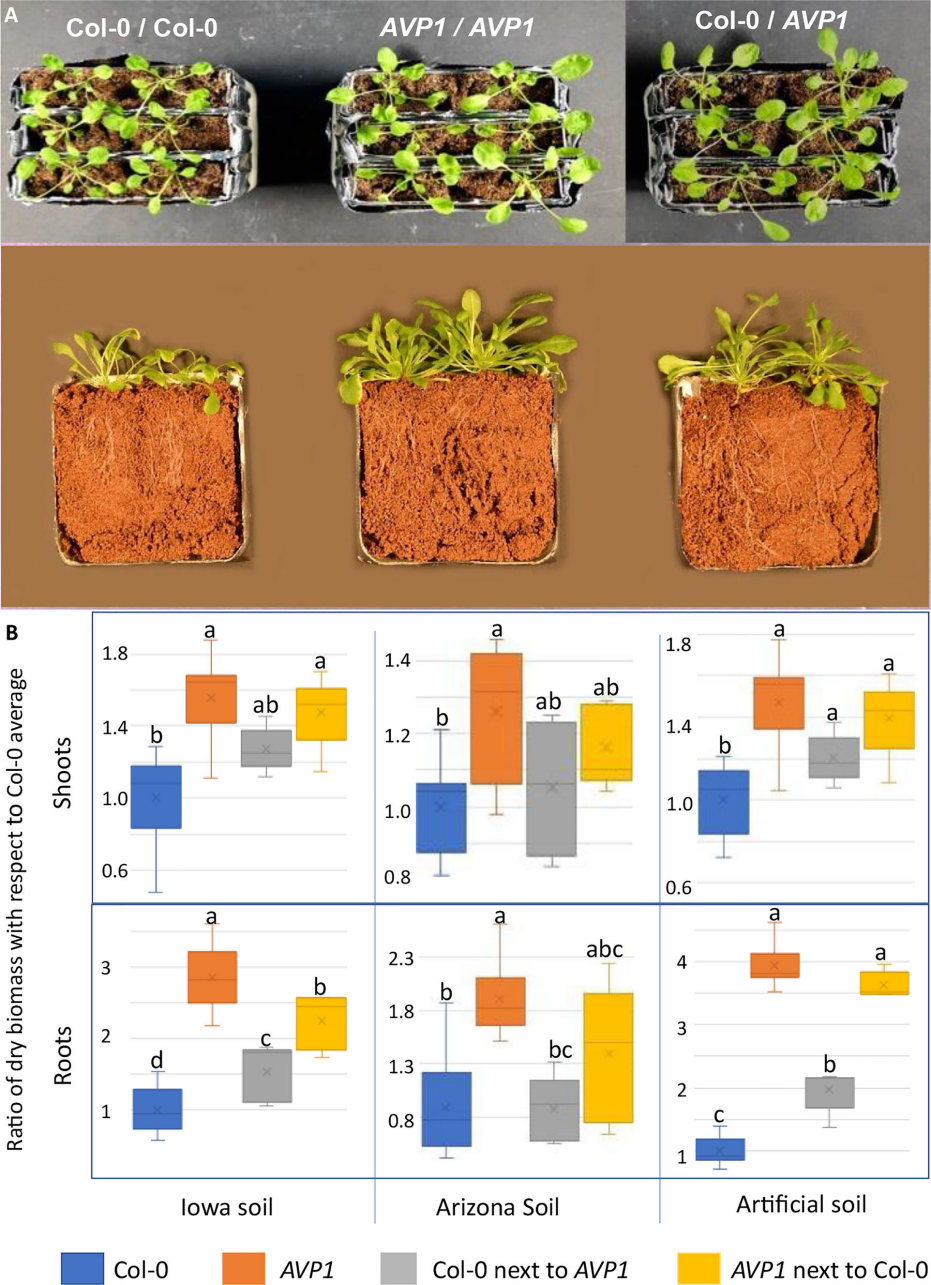
Phenotypic transfer between wild‐type *Arabidopsis thaliana* (Col‐0) and its derived transgenic *AVP1*. Photographs show phenotypic aspects of shoots (top row) as well as shoots and roots (second row) of plants grown as neighbor pairs on artificial soil. Genotypes are indicated in top row and apply to both rows. Bottom panel: root and shoot yield of the two genotypes grown as either homo‐ or heterogenetic pairs on different soils, with *n* = 14–16 per genotype presented as standard box‐and‐whisker plots. Different lowercase letters indicate significant differences among different treatments at the same evaluation time (*p* < 0.05).

The phenotype of *A. thaliana 35S::AVP1‐1* (with ubiquitous expression of H^+^‐PPase; hereafter, *AVP1*) could be partly transferred to nearby wild type plants (*A. thaliana* Col‐0; hereafter Col‐0), as seen qualitatively (Figure 1A) and quantitatively in Figure 1B. The transfer occurred with plants grown in artificial ceramic substrates (artificial soil) as well as in plants grown in two natural soils of differing potential fertility: an aridisol‐based garden soil from Arizona (Arizona soil), and a black agricultural soil from Iowa (Iowa soil).

When homogenetic plant pairs were grown in either of these substrates, *AVP1* genotypes always yielded significantly more biomass of shoots and roots than homogenetically grown Col‐0 pairs (post‐ANOVA *t*‐test corrected for multiple comparisons *p* < 0.001). But when grown as heterogenetic pairs, shoot and root mean yields of Col‐0 and *AVP1* plants were always intermediate to those of homogenetically grown pairs, although not always significantly different from both (Figure 1). However, at least one of the two genotypes in heterogenetic pairs differed in yield from those of its counterpart grown homogenetically or were not significantly different from those of their opposite genotype. In other words, there was a consistent significant loss of differentiation in Col‐0 yield when in the company of *AVP1*, as if the genotype of one plant was partly determining the phenotype of its neighbor.

Plant‐mediated microbiome engineering represents a compelling strategy that indirectly shapes the microbiome according to the phenotypic traits of the host plant. This approach assumes that the host's overall performance is indicative of the microbiome's effectiveness and the services it provides. Such indirect selection has been utilized for years across various agricultural systems, primarily as a means to overcome the technical challenges associated with directly monitoring microbiome functions (Dubey & Sharma, 2021). In our study, we observed that changes in the phenotypes of neighboring plants provide strong evidence for the efficient transfer of fitness traits from transgenic plants, further supporting the potential of this innovative strategy in enhancing plant resilience and productivity.

### 3.2 Extension of the mutant phenotype to the RAM

To establish if the mutant plant genotype affected its RAM, effectively extending the differential phenotype beyond the plant itself, we compared basic traits of Col‐0 and *AVP1* RAMs challenged by growth in soils that support divergent soil microbiomes (Figure S1), distinguishing microbiomes closely associated with the roots (Rhizosphere RAMs) and those loosely associated with the root systems (bulk root‐associated microbiomes [BRAMs]) (Barillot et al., 2013).

**FIGURE 2:**
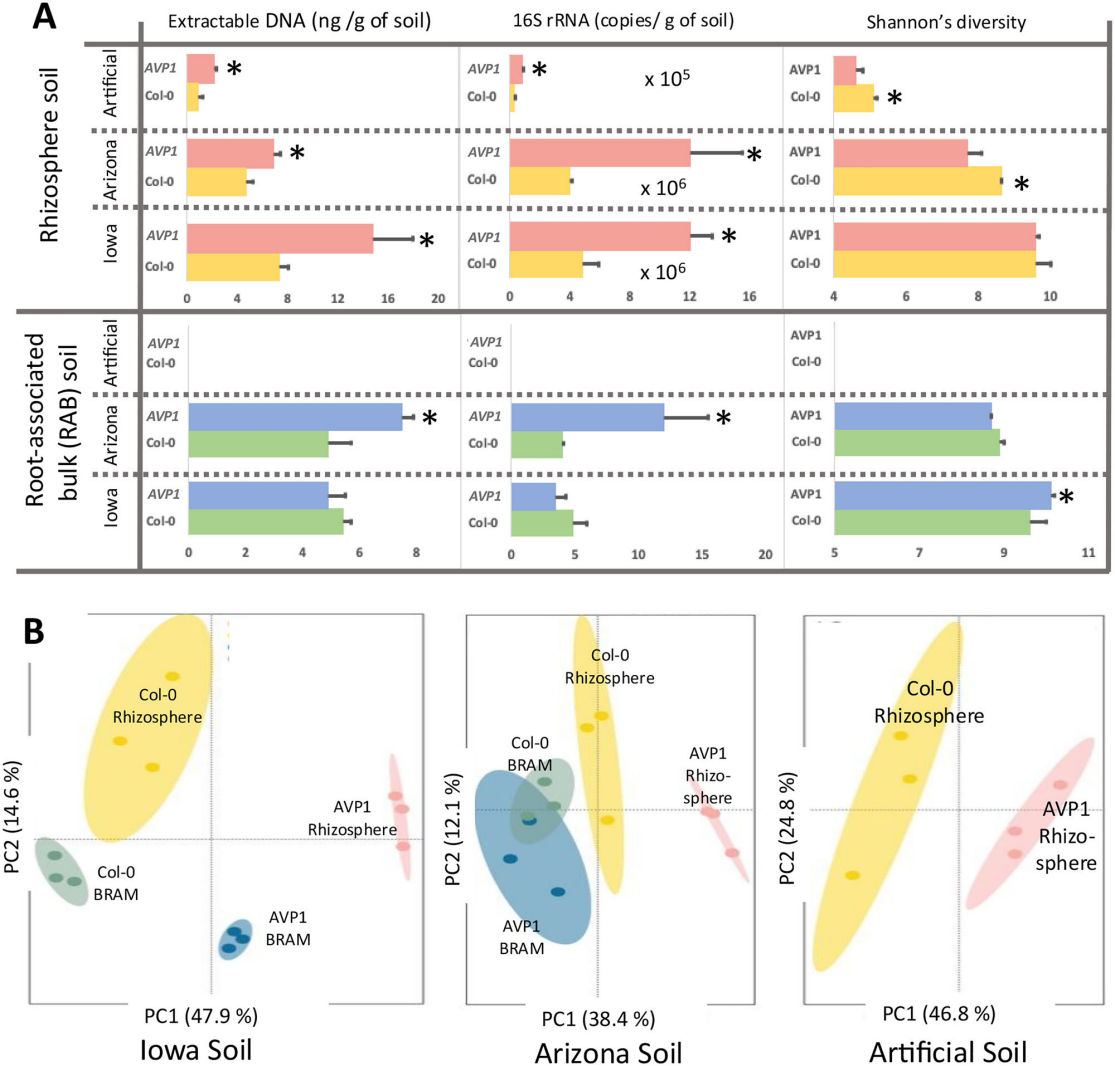
Extension of the plant genotype to the root‐associated microbiome. (A) Root‐associated microbiome (RAM) microbial biomass and diversity of mutant (*AVP1*) and wild‐type (Col‐0) plants grown in three different soils (*n* = 9 plants pooled into three measurements) for rhizosphere and root‐associated bulk (RAB) fractions; RAB was underdeveloped in artificial soil. (B) 16S rRNA gene‐based microbiome composition through principal component analyses (ellipses denote 95% CI; *n* = 9 plants pooled into three measurements) for plants grown in different soils. Explicit composition can be found in Figure S2. Asterisk indicates significant differences among different treatments at the same evaluation time (*p* < 0.05). Because RAB was underdeveloped in artificial soils, we could not perform direct measurements of DNA content (data missing), though it yielded enough DNA for sequencing.

The rhizosphere RAMs of *AVP1* were indeed significantly more populated than those of Col‐0, regardless of soil substrate, both in terms of extractable DNA and concentration of 16S rRNA gene copies determined by qPCR (Figure 2A). While both measures of biomass have their shortcomings, as plant‐derived DNA could have contributed to these tallies, and total 16S might be dependent on community composition (Gonzalez‐de‐Salceda & Garcia‐Pichel, 2021), sequencing analyses showed that plant plastid sequences were always a very minor proportion of all reads (Figure S2) that could not have affected the overall outcome of the comparison and the outcome was consistent for both, making our conclusions the most parsimonious explanation. Rhizosphere RAMs were also significantly less diverse in Arizona and Artificial soils, based on amplicon sequence analyses of 16S rRNA genes, although not so in naturally species‐rich Iowa soils.

Further, based on compositional similarity analyses, rhizosphere RAM bacterial communities (Figure 2B) of Col‐0 and AVPI differed significantly from each other, regardless of the soil substrate they were grown on. Thus, the differential phenotype associated with the H^+^‐PPase overexpressing mutation robustly extended to the rhizosphere microbiome across a variety of soils that contain highly divergent RAMs.

However, changes in community composition between plant genotypes did not involve the same bacterial types in the different soils: while in Arizona soils *AVP1* recruited more Proteobacteria and less Actinobacteria than Col‐0, in Iowa soils it recruited more Bacteroidetes and Verrucomicrobia to the detriment of Actinobacteria. Yet in artificial soils, which were initially devoid of a soil microbiome, and in which the RAM had to develop from environmental bacteria in the incubation chamber aerobiome, AVPI preferentially recruited Actinobacteria and Bacteriodetes to the detriment of Proteobacteria (Figures S2 and S3).

Apparently, the differential selection of microbial types is exerted on the available biodiversity (Liu et al., 2019; Song et al., 2022; Zhalnina et al., 2018), which differed significantly among soils. The effects seen on rhizosphere RAMs in some cases extended to the BRAMs (Figure 2A), either in composition or in abundance, or both, except in the artificial soil, where a BRAM was underdeveloped.

### 3.3 Microbiome blending and phenotype transfer agency

**FIGURE 3:**
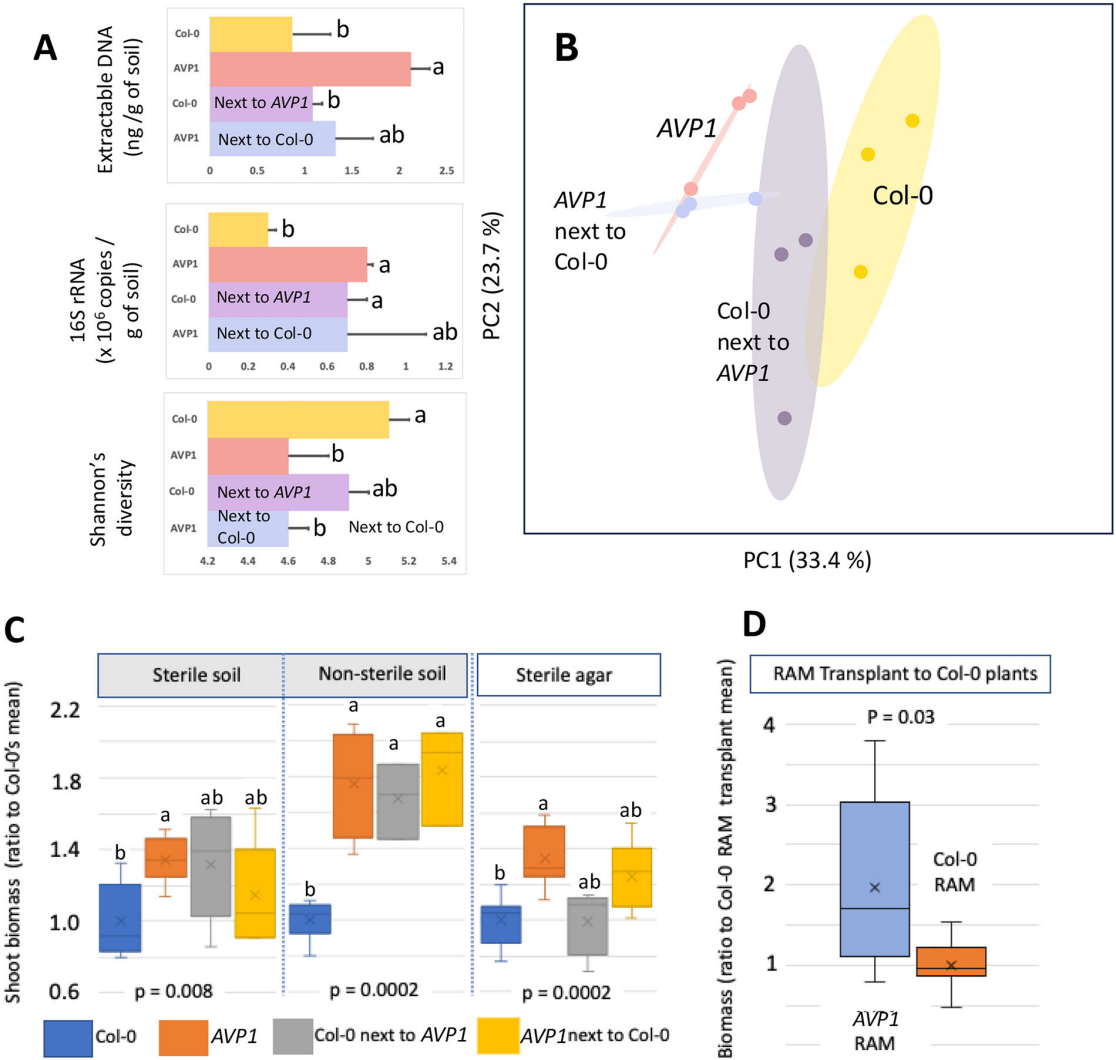
Transferability of plant and root‐associated microbiome (RAM) phenotypes between plants, and agency of the microbiome. (A) General traits of rhizosphere microbiomes from plants grown as either homogenetic or heterogenetic pairs on artificial soils (18 plants pooled into *n* = 6 measurements). (B) community composition of their rhizosphere RAMS (*n* = 3 measurements). (C) Effect of substrate sterility during the entire growth period on plant phenotypes (*n* = 6–8). (D) Effects on phenotype of Col‐0 plants (*n* = 8) by experimental RAM transplant from either Col‐0 or AVPI plants. Different lowercase letters indicate significant differences among different treatments at the same evaluation time (*p* < 0.05).

In these Artificial soils, however, it was possible to visibly trace and separate the roots according to plant genotype when Col‐0 and *AVP1* were grown as heterogenetic pairs, enabling an assessment of the transferability of RAM characteristics. Plants grown with the opposite genotype developed RAMS that were intermediate in composition (Figure 3B), and often in diversity and population density compared to those grown with plants of their own genotype (Figure 3A). The rhizosphere RAM phenotypes, like the plant phenotypes, were thus also partly and naturally transferable. In fact, when plants were grown without microbes (in Artificial soils or agar medium, grown fully within sterile chambers), phenotypic differences between wild‐type and transgenic were starkly diminished, and phenotypes failed to transfer (Figure 3C).

This indicated that while the genotype was partly and directly responsible for the phenotype, the magnitude of the differences was directly traceable to the presence of a microbiome. To test more strictly the agency of the RAMs in this phenomenon we performed a microbiome transplant experiment wherein RAMs from *AVP1* and Col‐0 grown separately were collected and inoculated in equal amounts (on a microbial cell basis) onto artificial sterile soils on which Col‐0 plants were then grown. Plants grown in the soil inoculated with AVPI RAM displayed *AVP1*‐like phenotypes, close to doubling their biomass (Figure 2D) over those inoculated with Col‐0 RAM, an effect that involved both shoots (*p* = 0.04) and roots (*p* = 0.006).

### 3.4 Mechanisms of plant/microbiome interaction

**FIGURE 4:**
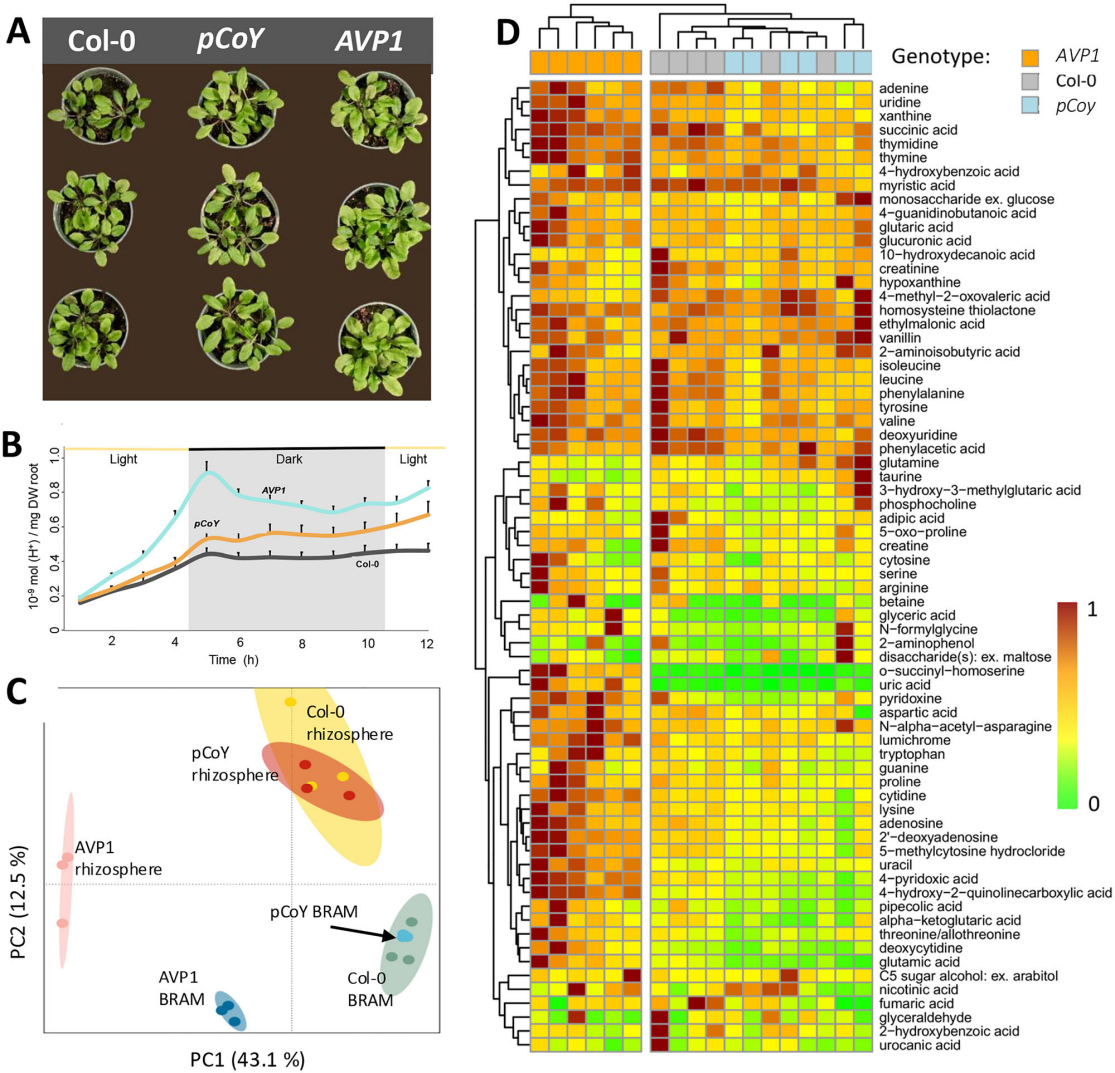
Mechanistic aspects of phenotype extension to the root‐associated microbiomes. A: phenotypes of Col‐0 (wild type) and genetically modified plants expressing the transgenic pyrophosphatase in all tissues (*AVP1*) or in phloem cells only (*pCoY*), where average yields are *AVP1* > *pCoY* > Col‐0 (Figure S4). (B) differential root acidification capacity of each genotype. (C) 16S rRNA‐based composition of their root‐associated microbiomes (RAMs) through principal component analyses. (D) Metabolomic profile of root exudates of the three plant genotypes, depicted as a heat map of relative concentration (each column pools *n* > 3 plants).

To probe how the plant genotype might be made extensive to the RAM, we made use of a second mutant plant (*pCoYMV::AVP1*; *pCoY* hereafter), which overexpresses the H^+^‐PPase in companion cells and sieve elements of the plant's phloem (Khadilkar et al., 2016). The yields of *pCoY* plants were not significantly different from Col‐0's (Figure 4A and Figure S4), but *pCoY* still acidified the soil more than the wild type, albeit not as strongly as AVP1 (Figure 4B). We found that the composition (Figure 4C) of *pCoY* RAMs did not differ from that of Col‐0′s, in either rhizosphere or BRAM fractions, but was significantly different from those of *AVP1*. If acidification were the major factor driving RAM community changes in transgenics, one would have expected some shifts in *pCoY*’s RAM similar to those detected for *AVP1*, but we did not detect any (Figure 4C). We then assessed a second potential driver: root exudates. We determined the root exudate profiles, or exometabolome (Baran et al., 2015), of all three genotypes when grown under sterility on agar‐solidified medium. The exometabolome of replicate genotypes (Figure 4D; see Supporting Information S1 for identification details) were most self‐similar, and those of *pCoY* were indistinguishable from those of Col‐0. A set of 18 compounds were excreted by *AVP1* preferentially (ANOVA + Tukey's test, *p* < 0.05), all nitrogenous metabolites, with nitrogenous bases, amino acids and their derivatives being prominent, consistent with the shift toward increase nitrogen metabolism typical of *AVP1* (Gonzalez et al., 2010).

This differential excretion is likely to exert a preferential functional enrichment on available soil microbial biodiversity. To test this, we conducted a comparative analysis of the rhizosphere RAM metagenomes of COL‐0 and *AVPI* plants, interrogating the metagenomes with specific functional questions or hypotheses derived from the existing evidence. Indeed, consistent with *AVP1*’s nitrogenous root excretions, its RAM metagenome was comparatively enriched in uptake genes for reduced nitrogen and depleted in those for nitrate uptake, while the proportion of genes for universal functions such as cell division was not different (Table S1). We then tested the hypothesis that microbiome‐produced plant hormones may be the link between microbiome and plant phenotype.

**FIGURE 5:**
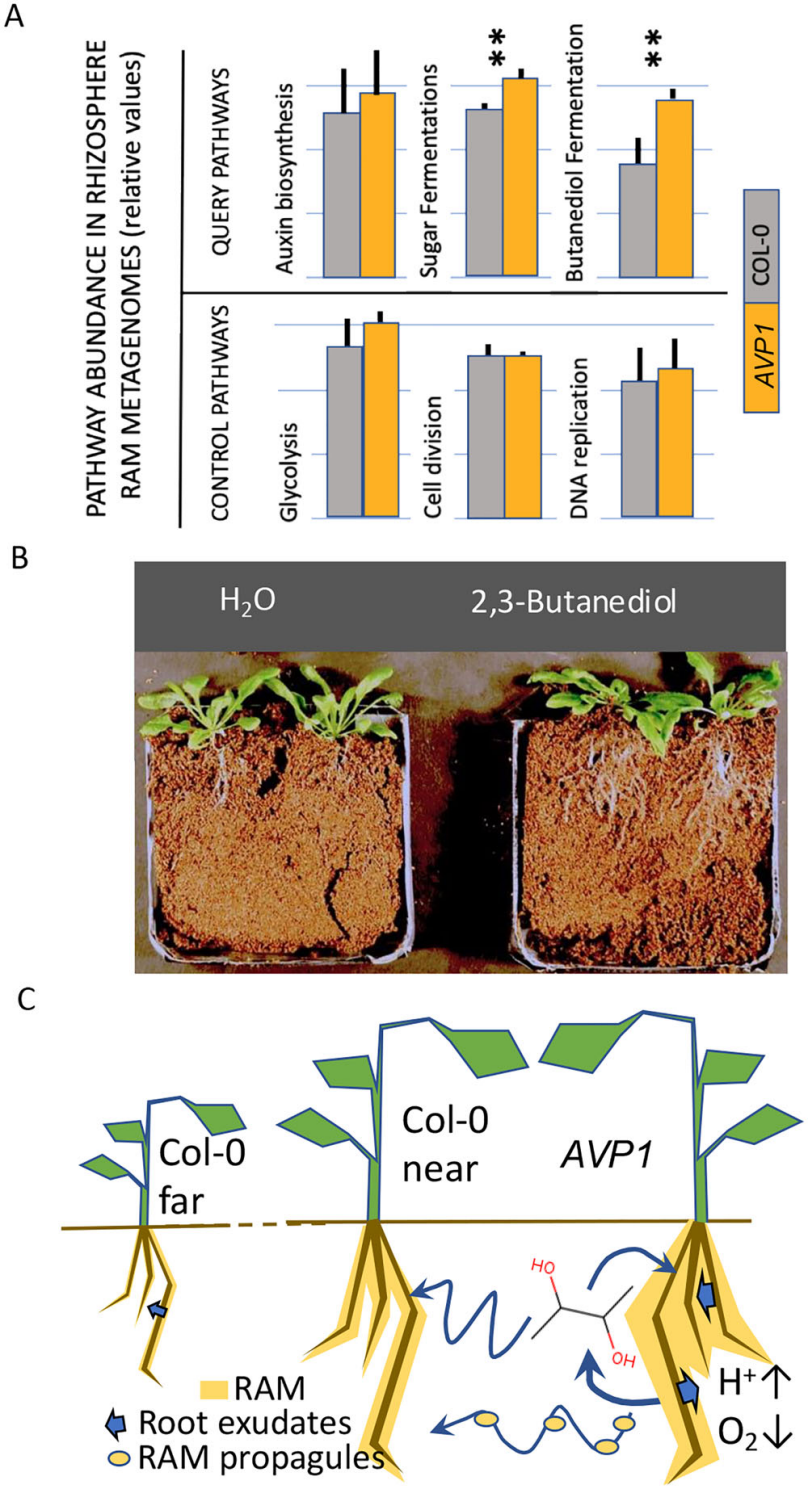
Mechanistic aspects of root‐associated microbiome (RAM) influence on plant phenotype. (A) Enrichment in proportional abundance of query and control pathways in the metagenome of rhizosphere RAMs. (Full results and statistics are in Table S1). (B) Effect of exposure to 250 µM 2,3‐butanediol on root development of Col‐0 plants. (C) mechanistic model for plant‐microbe interactions at the base of phenotype enhancement and transferability. Asterisk indicates significant differences among different treatments at the same evaluation time (*p* < 0.05).

Because of the abundance of tryptophan in the exudates of *AVP1*, a candidate was auxin (indole acetic acid), but the genes for its synthesis were not significantly enriched in *AVP1* RAMs (Figure 5A). Alternatively, the volatile 2,3‐butanediol, a product of bacterial fermentation, can also act as a growth, inducing hormone (Ryu et al., 2003). Indeed, key genes for major fermentation pathways were enriched in *AVP1* RAMs, consistent with a denser RAM that may more easily become oxygen limited. Among them, 2,3‐butanediol fermentation was the most enriched (Figure 5A), consistent with it being a preferred bacterial fermentation pathway under acidic conditions (Van Houdt et al., 2007).

The microbial fermentation of 2,3‐butanediol involves the stepwise conversion of pyruvate to α‐acetolactate via *alsS*, then to acetoin via *alsD*, and finally to 2,3‐butanediol through *bdhA*‐encoded acetoin reductase (Cruz Ramos et al., 2000; Nicholson, 2008). Volatile intermediates such as acetoin and 2,3‐butanediol function as microbial signals that influence plant physiology, including induction of stomatal closure and systemic resistance through nitric oxide and hydrogen peroxide (H_2_O_2_) signaling within guard cells (Wu et al., 2018). These responses intersect with abscisic acid and salicylic acid‐mediated defense pathways, linking microbial volatiles to core plant immune and growth regulation networks. Beyond signaling, 2,3‐butanediol exposure alters primary metabolism by elevating levels of key organic acids—such as aconitic, malic, and glycolic acids—associated with enhanced tricarboxylic acid cycle activity and respiration (Shi et al., 2018).

This metabolic reprogramming supports energy production and stress resilience and is accompanied by the accumulation of specific sugars and sugar acids, metabolic patterns associated with γ‐aminobutyric acid signaling and increased abiotic stress tolerance (Z. Li et al., 2016; Shi et al., 2018). Collectively, these effects highlight the role of 2,3‐butanediol in modulating plant energy homeostasis and defense, contributing to improved growth and enabling phenotype transfer to neighboring plants.

Metagenomics thus indirectly implicated 2,3‐butanediol production as the missing link. To test this strictly, we grew wild type plants in the presence of 2,3‐butanediol. This indeed resulted in *AVP1*‐like, high yield phenotypes not only in shoots as previously shown (Ryu et al., 2003), but also in root development (Figure 5B).

The demonstrable agency of the RAM as a determinant of plant phenotype is crucial in promoting plant‐to‐plant phenotype transfer because, while it partakes of the increase vigor imposed by the plant genetic mutation, it is not physically as constrained in space as plant tissues are. Clearly the transgenic plants supported a much larger root microbiome: A fourfold increase in root biomass (Figure 1B) compounded with a threefold difference in microbiome concentration (Figure 2A) implies that the rhizosphere RAM of *AVP1* transgenic plants were close to an order of magnitude larger than that of the wild type's. Given the transgenic RAM's prominence, and its differential functional character, it is in hindsight perhaps not so surprising that it can elicit plant phenotype sharing.

In our study, wild‐type Arabidopsis plants exposed to microbiomes enriched in 2,3‐butanediol—fermenting bacteria or to exogenous 2,3‐butanediol exhibited enhanced root and shoot growth that mimicked transgenic high‐yield phenotypes. This supports a mechanistic model in which microbial 2,3‐butanediol acts as a volatile plant signal, promoting growth through both stomatal regulation and transcriptional reprogramming of developmental and stress‐related pathways. Previous transcriptomic analyses confirm that 2,3‐butanediol can upregulate genes associated with cell wall modification (e.g., *expansin1* and *expansin3*), carbon and nitrogen metabolism (e.g., isocitrate lyase, nitrate reductase‐like genes), and hormone regulation (e.g., ARR‐A cytokinin response regulator), contributing to improved biomass production and heat stress resilience (Shi et al., 2018)

Mechanistically (see diagram in Figure 5C) high numbers of bacteria increase their colonization potential of neighboring roots on the one hand, and, on the other, their selective production of hormone‐like 2,3‐butanediol, which is volatile, provides a logical combined basis for phenotype transferability. Our current mechanistic model is thus consistent with the findings, although further inquiries using specific bacterial isolates would be welcome. We find it remarkable, however, that such proximal phenotype transfer could occur in a single generation. It materialized much faster than other potential secondary effects based on plant evolutionary processes (Lau & Lennon, 2012) or transgene introgression (Stewart et al., 2003).

By extension of this mechanistic explanation, one could also expect that it may result in cumulative effects after repeated cycles of plant growth, as the soil microbiomes become progressively more enriched in 2,3‐butanediol fermenters, perhaps also extending its spatial range.

For future applications, it is crucial to recognize the significant impact that the delivery system has on plant responses. For instance, applying sucrose directly to the soil failed to replicate the effects observed from enhanced sugar secretion by plants. In this scenario, the species composition of the soil microbiome remained unchanged compared to the control soil that did not receive sugar supplements. This finding underscores the importance of the sugar transfer pathway to microorganisms. It appears that sugars secreted from root epidermal cells are preferentially absorbed by microbes in close association with the roots, potentially leading to shifts in microbial community composition and behavior that could influence the entire soil microbiome (Song et al., 2022).

Moreover, 2,3‐butanediol, a volatile compound associated with plant signaling pathways, may also play a role in microbiome recruitment (Kong et al., 2018; Park et al., 2022). This suggests that the recruitment of beneficial microbes might be part of a secondary response arising from altered plant metabolic activity. Understanding these dynamics could enhance our strategies for optimizing plant‐microbe interactions and maximizing agricultural productivity.

While it's well established that plant genotypes influence microbial communities, most studies have focused on broad taxonomic changes or mutualistic outcomes like nutrient uptake and disease resistance (Berg & Smalla, 2009; Lebeis et al., 2015; Song et al., 2022). Here, we show that AVP1 transgenic plants go further—reshaping not just the composition but also the function of the RAM in ways that trigger phenotypic changes in neighboring, non‐transgenic plants. This effect is driven by specific shifts in the exometabolome, particularly a marked enrichment in nitrogen‐rich compounds, which selectively recruit microbial groups such as 2,3‐butanediol producers. In contrast, wild‐type and phloem‐specific overexpression lines showed similar exudate profiles and lacked this microbiome reprogramming effect.

These findings reveal that both the quantity and quality of exudation are critical for microbiome engineering and suggest a new strategy: tailoring root exudates—via genetic or chemical means—to assemble beneficial microbial consortia in situ. By enabling transgene‐independent trait transfer through microbiome modulation, this work offers a compelling alternative to traditional breeding or inoculation‐based approaches.

## 4 CONCLUSIONS

Our research traces the transfer of transgenic phenotypes to wild‐type *Arabidopsis* plants to the agency of their RAM, highlighting the microbiome as a key determinant of plant phenotype. Notably, this finding suggests that the genotype of one plant can significantly modify the expression of another plant's genotype, which raises important considerations for the deployment of transgenic plants. Specifically, some of the vigor exhibited by transgenic plants may be redirected toward supporting the growth of neighboring plants, potentially including undesired species.

While these logical predictions warrant further investigation, the specific interaction mechanisms observed between *AVP1* transgenic roots, and their associated microbiome suggest that such phenotype transfer is unlikely to be a universal trait across all transgenic varieties. Given these insights, it is advisable to incorporate assessments of RAM effects into the risk‐benefit evaluations of transgenic plants.

On a more optimistic note, our findings, particularly from the RAM transplant experiments, open up exciting possibilities for developing plants with desirable growth characteristics akin to those of transgenics, without the need to introduce transgenic genotypes directly into the field. Instead, this approach could focus on utilizing preselected beneficial RAMs, potentially offering a sustainable and innovative pathway for enhancing agricultural productivity.

## AUTHOR CONTRIBUTIONS

**Ferran Garcia‐Pichel**: Conceptualization; formal analysis; investigation; methodology; project administration; resources; supervision; writing—original draft; writing—review and editing. **Júlia Farias**: Formal analysis; investigation; methodology; writing—review and editing. **Vanessa Fernandes**: Investigation; methodology. **Daniel Roush**: Data curation; formal analysis. **Tami L. Swenson**: Data curation; formal analysis. **Suzanne M. Kosina**: Data curation; formal analysis. **Trent R. Northen**: Data curation; formal analysis; writing—review and editing. **Huansheng Cao**: Data curation; formal analysis. **Samual Jaunin**: Data curation; formal analysis. **Raju Kandel**: Data curation; formal analysis; investigation; methodology. **Roberto Gaxiola**: Formal analysis; investigation; methodology; supervision; writing—review and editing.

## CONFLICT OF INTEREST STATEMENT

The authors declare no conflicts of interest.

## Supporting information



Supplemental material

## Data Availability

Raw data files have been deposited in the JGI Genome Portal and are available for download here: https://genome.jgi.doe.gov/portal/201Tratabolomics_FD/201Tratabolomics_FD.info.html under project ID # 1266724.
