# Supplementary material for: Swift microbiome‐mediated phenotype transfer from transgenic plants
Source: J Environ Qual. 2025 Aug 15;54(6):1368–82. doi: 10.1002/jeq2.70070 (PMC12593279; doi:10.1002/jeq2.70070)
Supplement: Supplementary file 1 — Supplemental material [file JEQ2-54-1368-s001.docx]

Supplemental Material


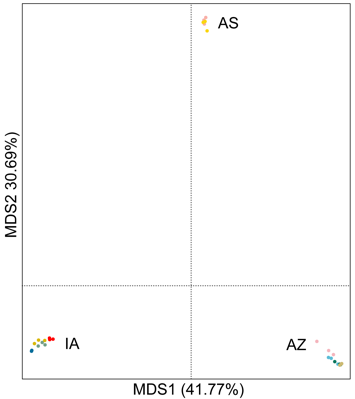
**Additional Files**

Fig. S1. Compositional differences among RAMs in soils used for experimentation, based on 16S rRNA sequences, including determinations on rhizosphere RAM, and BRAM, for Col-0 and AVP1 plants. Differences among soils are much larger than those between plants in the same soils. AS: artificial soil. AZ: Arizona soil. IA: Iowa soil. Each row represents a unique amplicon sequence variant (ASV) identified through 16S rRNA sequencing. Statistical comparisons were performed to evaluate the relative abundance of each ASV between genotypes under each substrate condition.

Fig S2. RAM community composition at the Phylum level, based on 16S rRNA sequences and bioinformatic placement, for different RAM fractions, plant genotypes, genotypic neighbor, or soil substrates, as indicated in the left axis. Absolute values are in the bar graphs to the left, and relative composition to the right. Each condition has 3 composite samples independently processed with respect to incubation and sampling. Because RAB was underdeveloped in artificial soils, we could not perform direct measurements of DNA content (data missing), though it yielded enough DNA for sequencing.

Fig. S3. Volcano plots of relative abundance for individual 16S rRNA gene bacterial sequences between the rhizosphere RAMS of *AVP1* and Col-0, when grown on different substrates. Sequences the differential abundance of was significantly different are denoted by red dots.

Fig.S4. Biomass yields for wild type (Col-0) *A. thaliana* plants and the derived proton pyrophosphate transgenics *pCoy* and *AVP1*, relative to the yield of the wild type.

Fig. S5. Key for accessing metabolomics LCMS raw and processed data as a zip file after creating a user account from

<https://genome.jgi.doe.gov/portal/201Tratabolomics_FD/201Tratabolomics_FD.info.html>

under data ID numbers 1266727 and AP, respectively.

Supplementary Table S1
